# Reply to: Letter to Editor by Saravanan Sampoornam et al.

**DOI:** 10.1111/jcmm.70465

**Published:** 2025-03-03

**Authors:** Shukoofeh Torabi, Faezeh Shekari, Mustapha Najimi, Massoud Vosough

**Affiliations:** ^1^ Department of Applied Cell Sciences, Faculty of Medicine Kashan University of Medical Sciences Kashan Iran; ^2^ Department of Stem Cells and Developmental Biology Cell Science Research Center, Royan Institute for Stem Cell Biology and Technology, ACECR Tehran Iran; ^3^ Laboratory of Pediatric Hepatology and Cell Therapy Institute of Experimental and Clinical Research (IREC), UCLouvain Brussels Belgium; ^4^ Department of Regenerative Medicine, Cell Science Research Center Royan Institute for Stem Cell Biology and Technology, ACECR Tehran Iran; ^5^ Experimental Cancer Medicine, Institution for Laboratory Medicine Karolinska Institute Stockholm Sweden

We appreciate the comments raised in the letter to the editor [1] which contribute to a deeper understanding of extracellular vesicle characterisation and mechanisms of action. We strongly believe that enhanced transparency is crucial for the reproducibility of our findings and for advancing research in this field [2].

In our study, the conditioned medium of WJ‐MSCs was collected and then concentrated 10 times using a tangential flow filtration system and centrifuged at a low speed (3000 × *g* for 10 min at 4°C) to remove cellular debris from the concentrated medium. This partially clarified medium was then subjected to centrifugal forces in the range of 20,000–110,000 × *g* to isolate two subpopulations: EV20K and EV110K [3]. Regarding EV characterisation, we believe that the most authoritative references for validation parameters are MISEV2018 and MISEV2023 guidelines [4]. Accordingly, we adhere to these guidelines to ensure robust and reproducible results. These guidelines recommend that one or a combination of the following methods can be used to quantify EVs (particle number, protein and/or lipid content) as well as to directly measure their size [nanoparticle tracking analysis (NTA) and dynamic light scattering (DLS)] [4]. Each particle size analysis method has some advantages and drawbacks. For instance, even though NTA enables EV characterisation by both size and concentration, several factors can affect NTA results, including lipoprotein contamination, the number of freeze/thaw cycles, sample filtration, video length and particle density per frame [5]. Since DLS is more user‐friendly and provides results more quickly than other methods [6], EV size distribution was determined using DLS, whereas EV concentration was estimated using total protein quantification.

For labelling EVs, several dyes with unique characteristics can be used, including PKH, DiD and calceins. PKH and DiD are lipophilic dyes that exhibit a strong fluorescent signal when incorporated into EV membranes. Despite the excellent stability of both dyes for long‐term tracking, some challenges are associated with these dye‐binding assays. These challenges include lipoprotein contamination in EV samples, binding non‐specifically to all cell membrane fragments and non‐intact EVs, dissociation from EVs into blood and serum components and false positive signals due to self‐aggregation and the formation of micelles by dyes that have the same size and morphology similar to EVs [7, 8]. To overcome these challenges, the cellular uptake and integrity of EVs have been evaluated using membrane‐impermeable fluorescent dyes like calceins. These fluorescent dyes can only identify metabolically active and intact vesicles since they require intracellular esterases to release calcein and become fluorescent [9]. EV labelling procedures were performed for calcein as previously described [10].

Complex biological supplements for cell culture, such as human platelet lysate (hPL), contain their own EVs, which can significantly affect cell physiology, EV release and overall experimental outcomes. The complete removal of these EVs is often impossible; moreover, attempts to remove them may also compromise their vital biological roles [11]. Recognising these challenges, the ISEV Task Force on conditioned medium‐derived EVs (CCM‐EVs) has recommended that all relevant parameters be reported transparently to ensure reproducibility. In our study, we have followed these guidelines to maintain clarity and reliability in our results. Hence, all experiments were conducted with the same batch of hPL at a consistent concentration for both control and EV‐treated groups to create a steady‐state conditions in evaluating the biological effects of EVs on the behaviour of recipient cells.

Results of our previous comprehensive quantitative proteomic analysis of MSC‐derived EVs showed a high abundance of proteins involved in translation and metabolic pathways, such as oxidative phosphorylation, and a low abundance of proteins involved in inflammation and cell death in the EVs isolated by high‐speed centrifugation (HS; 20,000*g*) [12]. Since our other previous studies [13, 14, 15] also supported the functionality of EV20K in inducing an immunosuppressive response, we investigated the potential of this subset of EVs to serve as an off‐the‐shelf, GMP‐compatible product that might provide a feasible way to educate monocytes into a novel subset of alternatively activated macrophages in this study. It is widely acknowledged that in vivo conditions bring a notable level of complexity that can greatly influence the outcomes. As part of our future research, we plan to test EV‐educated macrophages for their anti‐fibrotic properties in in vivo models to gain a deeper understanding of the possible interactions within a physiologically relevant setting.

## Author Contributions

**Shukoofeh Torabi:** conceptualization (equal), data curation (equal), investigation (equal), writing – original draft (equal). **Faezeh Shekari:** validation (equal), writing – review and editing (equal). **Mustapha Najimi:** validation (equal), writing – review and editing (equal). **Massoud Vosough:** validation (equal), writing – review and editing (equal).

## Conflicts of Interest

The authors declare no conflicts of interest.

## Data Availability

The authors have nothing to report.
